# Increased Circulating Th17 but Decreased CD4^+^Foxp3^+^ Treg and CD19^+^CD1d^hi^CD5^+^ Breg Subsets in New-Onset Graves' Disease

**DOI:** 10.1155/2017/8431838

**Published:** 2017-11-13

**Authors:** Jing Qin, Jin Zhou, Chenling Fan, Na Zhao, Yongping Liu, Shuo Wang, Xuejiao Cui, Mingshi Huang, Haixia Guan, Yushu Li, Zhongyan Shan, Weiping Teng

**Affiliations:** ^1^Department of Endocrinology and Metabolism, Institute of Endocrinology, Liaoning Provincial Key Laboratory of Endocrine Diseases, The First Affiliated Hospital of China Medical University, Shenyang, Liaoning, China; ^2^Department of Endocrinology, Affiliated Yantai Yuhuangding Hospital of Qingdao University Medical College, Yantai, Shandong, China

## Abstract

Th17 and regulatory lymphocyte subsets such as Tregs and Bregs have been reported to play important roles in autoimmune diseases. The aim of this work was to perform quantitative studies of circulating Th17, Tregs, and Bregs in patients with new-onset Graves' disease (GD). Twenty GD patients and 20 healthy controls were involved in this study. Blood samples were taken for flow cytometry detection of CD4^+^IL-17^+^ Th17, CD4^+^Foxp3^+^ Tregs, and CD19^+^CD1d^hi^CD5^+^ Bregs and meanwhile, for real-time PCR measurement of gene expressions of ROR*γ*t, IL-17 and IL-10. The proportions of Tregs and Bregs as well as the Foxp3 gene expression but not IL-10 were significantly decreased in GD group compared with the healthy controls. The frequency of Th17 together with the gene expressions of ROR*γ*t and IL-17 were significantly increased in the GD group. Furthermore, the Th17/Treg ratio was also significantly higher in GD group. A significant positive correlation between Th17 and TSAb (*r* = 0.656, *p* < 0.001) but significant negative correlations between Treg/Breg and TSAb (*r* = −0.339, *p* = 0.032; *r* = −0.759, *p* < 0.001) were identified among the participants. This study indicated that increased Th17 and impaired Treg responses, along with a decreased number of CD19^+^CD1d^hi^CD5^+^ Breg cells, were involved in GD pathogenesis.

## 1. Introduction

Graves' disease (GD) is one of the most common organ-specific autoimmune diseases. It is characterized by hyperthyroidism and specific thyroid autoantibodies against thyroid-stimulating hormone receptor (TSHR) and is also commonly accompanied by autoantibodies against thyroglobulin (TG) and thyroid peroxidase (TPO).

Despite the environmental and genetic factors, the aetiology of GD is partly due to the breakdown of immune self-tolerance to thyroid autoantigen TSHR, thus leading to autoreactive T and B cells. It has been widely accepted that the exaggerated T helper (Th) 2 response plays a pivotal role in GD development [1–3]. However, the Th2 theory cannot explain all conditions. Recently, researchers have recognized that two other CD4^+^ T cell subsets and one B cell subtype, namely, the Th17 and regulatory T (Treg) cells, along with regulatory B (Breg) cells, may participate in GD pathogenesis [4–9].

Th17 cells, a newly identified CD4^+^ T cell subgroup, function by secreting their signature cytokine, interleukin-17A (IL-17A, also called IL-17). Numerous studies have demonstrated the pathogenic role of Th17 cells in autoimmune diseases, including inflammatory bowel disease, rheumatoid arthritis (RA), psoriasis, and asthma [10–13]. Treg cells, characterized by CD4, CD25, and transcription factor forkhead box P3 (Foxp3) expression, are reported to help maintain immune self-tolerance and to suppress autoreactive T and B cells through cell-to-cell contact or the secretion of regulatory cytokines such as interleukin-10 (IL-10) and transforming growth factor-*β* (TGF-*β*) [14]. In a previous study, we explored the quantitative changes of Th17 and Treg populations in a murine model of Graves' hyperthyroidism [4]. We wanted to know whether the tendency is similar in patients with new-onset GD. Recently, studies have been conducted in individuals by other groups, yet the outcomes have been inconsistent [6–8]. The question of how the Th17 and Treg populations are modulated in new-onset GD patients remains to be clarified.

It has long been recognized that B cells not only can play “bad guys” in autoimmune diseases to produce autoantibodies or to present autoantigens to T cells but also can also act as “good guys,” exhibiting immune-regulatory capacity that is mainly mediated by IL-10 secretion. The B cell subset with the surface marker combinations of CD1d^hi^CD5^+^, which is also called B10 due to their competence of secreting IL-10, has been widely reported in mouse. In some murine models of autoimmune diseases, adoptive transfer of these B10 cells will limit the disease development [15, 16]. However, very few studies about this B cell phenotype have been performed in humans, and few studies have explored the quantitative changes in the circulating CD1d^hi^CD5^+^ Breg subset in diseases such as RA and tuberculosis [17, 18]. Nevertheless, little is known about this B cell subset in GD patients.

Here, we described the proportions of Th17, CD4^+^Foxp3^+^ Treg, and CD19^+^CD1d^hi^CD5^+^ Breg cells and the mRNA expression levels of their main cytokines and transcription factors in the peripheral blood of new-onset GD patients and in healthy controls. Furthermore, we investigated the potential relationships between the differential expression of the lymphocyte subsets and the specific thyroid autoantibodies of GD.

## 2. Materials and Methods

### 2.1. Participants

Between May 2010 and August 2010, twenty newly diagnosed patients with GD (7 males and 13 females) and 20 healthy controls were recruited by the First Affiliated Hospital of China Medical University. The newly diagnosed GD patients had typical characteristics of hyperthyroidism: palpable, soft, and diffuse goitre confirmed by ultrasound examination, increased thyroid radioiodine uptake, elevated serum free thyroxine (FT4), very low thyroid-stimulating hormone (TSH), and a high thyroid-stimulating antibody (TSAb) level. Age- and gender-matched healthy controls with normal thyroid function (TSH: 0.5–2.0 mIU/L) and without goitre and any family history of GD were recruited during the same time. No participants presented clinical evidence of tumour and infectious or other autoimmune diseases or received antithyroid drugs. Those who had received immunosuppressive drugs, such as mannan peptide, lentinan, interferon, corticosteroids, and cyclophosphamide, within one month of the study, were also excluded. A written informed consent was obtained by all participants and the protocol was approved by Medical Ethics Committee of China Medical University.

### 2.2. Laboratory Tests

The participants' concentrations of serum TSH, FT4, thyroid peroxidase antibody (TPOAb), and thyroglobulin antibody (TgAb) were measured via chemiluminescent enzyme immunoassay using specific kits (IMMULITE, DPC, USA). For TSH, the normal range was 0.3–4.8 mU/L, and the intra-assay and interassay coefficients of variability (CVs) were 1.95–6.66% and 4.18%, respectively. For FT4, the detection range was 2.6–77.2 pmol/L, and the serum level of FT_4_ > 24.5 pmol/L was defined as elevated, while <10.3 pmol/L was defined as decreased; the intra-assay CV was 5.89%. For TPOAb and TgAb, the serum was diluted to 1 : 100 and was then tested using the IMMULITE analyser. The detection range of TPOAb was 10–1000 IU/ml, and TPOAb > 35 IU/ml was defined as positive, while the detection range of TgAb was 20–3000 IU/ml, and TgAb > 40 IU/ml was defined as positive; the intra- and interassay CVs were below 8.5%.

For TSAb activity, the serum IgG was first crudely purified with polyethylene glycol (PEG) and resuspended in hypotonic Hank's buffer. Next, the crude IgG was cocultured with CHO cells, which were transfected with TSH receptor (TSHR) to detect the IgG's effect on the cAMP production of CHO cells. The cAMP concentration was measured using a commercial radioimmunoassay, and the TSAb activity was calculated as as follows: 100 × (cAMP patient/cAMP euthyroid control). The cutoff value for positive TSAbs was set as the mean value + 3SD of the normal healthy controls, so the TSAb activity > 130.19% was defined as positive.

### 2.3. Preparation and Stimulation of Mononuclear Cells

Peripheral blood mononuclear cells (PBMCs) were prepared using a commercial LymphoPrep and density gradient centrifugation. Next, the cells were resuspended in RPMI-1640 (Gibco, USA) with 10% foetal bovine serum (FBS) (Gibco, USA), and the cell concentration was adjusted to 1*∗*10^7^/ml. PBMCs (10^6^/ml) were stimulated in 24-well plates with 50 ng/mL of phorbol myristate acetate (PMA) (Sigma, USA) and 1 *μ*g/mL of ionomycin (Sigma, USA) in the presence of brefeldin A (BFA) (BD, USA) at 37°C for 5 hours before flow cytometric staining and real-time polymerase chain reaction (RT-PCR).

### 2.4. Flow Cytometry Analysis

For regulatory B cell staining, anti-human monoclonal antibodies (mAbs) such as fluorescein isothiocyanate- (FITC-) conjugated anti-CD5 (clone:UCHT2), phycoerythrin- (PE-) conjugated anti-CD1d (CD1d42), and PerCP-Cy™5.5-conjugated anti-CD19 (HIB19) were used. For Th17 and Treg staining, a Human Th17/Treg Phenotype Kit (catalogue number: 560762) was used. All the antibodies and the kits were from BD Biosciences. We incubated 1*∗*10^6^ PBMCs with properly diluted fluorochrome-conjugated mAbs for surface staining for 30 minutes on ice and washed the cells twice with stain buffer (catalogue number: 554656, BD Biosciences). For intracellular IL-17 and Foxp3 detection, the cells were stained according to the manufacturer's instructions. Flow cytometric analysis was performed using a FACSCalibur (BD Biosciences) cytometer with CellQuest software, and the data were analysed using FlowJo 7.6.1 software.

### 2.5. RNA Extraction and Reverse Transcription-Quantitative PCR (RT-qPCR)

Total RNA was extracted from cells that had been stimulated for 5 hours using TRIzol reagent (Takara, Japan) according to the manufacturer's instructions. The RNA was transcribed into complementary DNA (cDNA) using a QPCR cDNA kit (PrimeScript™ RT Reagent Kit, Takara, Japan), and the transcripts were quantified using the SYBR® Premix Ex Taq™ II reagent (Takara, Japan) and a Lightcycler 480 Real-time PCR System (Roche, America). The amplification was performed for 40–45 cycles in a total volume of 20 *μ*l, and the relative expression levels were determined by normalizing each target to that of GAPDH. The primer sequences were designed as follows:* IL-10*, 5′-TCAAGGCGCATGTGAACTCC-3′ (forward primer) and 5′-GATGTCAAACTCACTCATGGCT-3′ (reverse primer);* Foxp3*, 5′-GCTTCATCTGTGGCATCATC-3′ and 5′-TGGAGGAACTCTGGGA ATGT-3′;* IL-17-A*, 5′-CAATCCCACGAAATCCAGGATG-3′ and 5′-GGTGGAGATTCCAAGGTG AGG-3′;* RORγt*, 5′-GTAACGCGGCCTACTCCTG-3′ and 5′-GT CTTGACCACTGGTTCCTGT-3′; and* GAPDH*, 5′-GAAATCCCATCACCATCTTCCAGG-3′ and 5′-GAGCCCCAGCCTTCTCCATG-3′. All samples were run in triplicate, and the data were analysed using LightCycler 480 Real-time Analysis Software.

### 2.6. Statistical Analysis

The analysis was performed using IBM SPSS Statistics 19.0 software. The data are expressed as the mean ± SD or median values with ranges. An unpaired two-tailed Student's *t*-test, one-way analysis of variance (ANOVA), or a nonparametric Mann–Whitney test was used for the comparisons between groups. The correlation analyses were conducted using the Pearson correlation test. A two-sided *p* value < 0.05 was considered significantly different.

## 3. Results

### 3.1. A Higher Proportion of Th17 Cells but Lower Proportions of CD4^+^Foxp3^+^ Treg and CD19^+^CD1d^hi^CD5^+^ Breg Cells in the Peripheral Blood of GD Patients Were Observed

The clinical characteristics of the participants are summarized in Table 1. As expected, no significant differences were noted in the age and gender distributions between the healthy controls and GD patients. The analysis of thyroid function in GD patients showed significantly higher serum FT4 but lower serum TSH levels than those in healthy controls (medians: 63.7 versus 16.0 (*p* < 0.001) and 0.004 versus 1.390 (*p* < 0.001), resp.). The activity of TSAb, the specific thyroid autoantibody produced in GD, was significantly higher in GD patients than in controls (median: 215.58 versus 100.00, resp., *p* < 0.001). In addition, the concentrations and positive rates of two other thyroid autoantibodies, TPOAb and TgAb, which are often elevated along with GD, were also significantly higher in the GD group than in the controls (median: 261.0 versus 10.0 (*p* < 0.001) and 20.0 versus 20.0, (*p* = 0.026), resp.). In summary, apart from thyroid functions and the thyroid autoantibody levels, the two groups were comparable.

**Figure 1:**
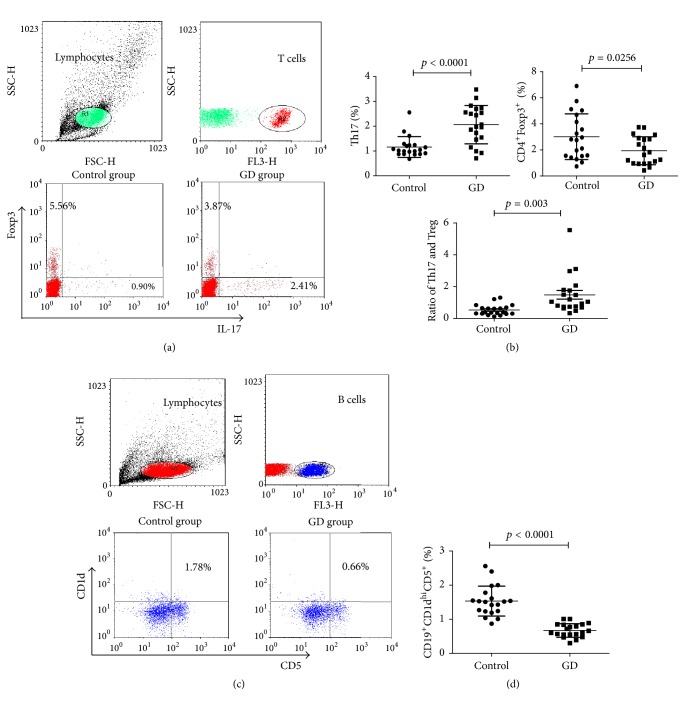
*The frequencies of circulating Th17 cells, CD4* ^+^ *Foxp3* ^+^ *Tregs, and CD19* ^+^ *CD1d* ^*hi*^ *CD5* ^+^ *Bregs and the ratio of Th17/Treg in patients and healthy controls*. PBMCs from participants' peripheral blood unstimulated or stimulated with PMA plus ionomycin were stained with a panel of fluorochrome-conjugated monoclonal antibodies against CD4, IL-17, and Foxp3 using Human Th17/Treg phenotype Kit or against CD19, CD5, and CD1d. In the three-color fluorescence cytometric analysis, cells were gated first on lymphocytes, then the CD4^+^ T cells were gated out for further IL-17^+^ and Foxp3^+^ T cell subsets analysis (a), and the CD19^+^ B cells were gated out for further CD1d^hi^CD5^+^ B cell subset analysis (c). The quantitative analysis of circulating CD4^+^IL-17^+^, CD4^+^Foxp3^+^ T cells and CD19^+^CD1d^hi^CD5^+^ B cells as well as the ratio of Th17/Treg were shown in (b) and (d). The quantitative analysis of individual values were shown as mean ± SD. A *p* value < 0.05 was considered statistical difference.

To evaluate the differences in circulating Th17, CD4^+^Foxp3^+^ Treg, and CD19^+^CD1d^hi^CD5^+^ Breg populations between GD patients and healthy controls, we next used three-color fluorescence cytometric analysis to analyse the percentages of CD4^+^IL-17^+^ and CD4^+^Foxp3^+^ T cell subsets and the CD19^+^CD1d^hi^CD5^+^ B cell subset in the PBMCs of all the participants (Figure 1). We observed a significantly higher percentage of Th17 cells in the GD group than in the controls (mean ± SD, 2.07 ± 0.77 versus 1.16 ± 0.42; *p* < 0.001). Moreover, the proportions of CD4^+^Foxp3^+^ Treg and CD19^+^CD1d^hi^CD5^+^ Breg subsets were significantly decreased in the GD group compared with those in the controls (mean ± SD, 1.95 ± 1.07 versus 3.02 ± 1.76, *p* = 0.0256; 0.66 ± 0.29 versus 1.53 ± 0.44, *p* < 0.001). In addition, we further estimated the ratio of Th17/Tregs and found that the ratio was higher in the GD group than that in the control group (mean ± SD, 1.477 ± 1.224 versus 0.526 ± 0.329, *p* = 0.003).

### 3.2. Higher Relative ROR-*γ*t and IL-17 but Lower Relative Foxp3 mRNA Expression Levels Were Detected in GD Patients

**Figure 2:**
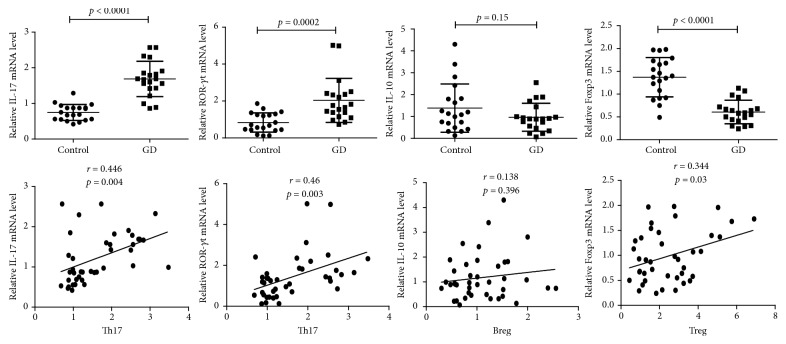
*Relative IL-17, ROR-γt, IL-10, and Foxp3 mRNA levels and their correlations with circulating Th17, Breg, and Treg cells in all participants*. Total RNAs were extracted from PBMCs from participants' peripheral blood. Then the RNAs were reverse-transcribed into complementary DNA(cDNA) and the transcripts were quantified using real-time PCR System. The relative expression levels were determined by normalizing each target to GAPDH. The quantitative analysis of individual values was shown as mean ± SD. The correlation analysis was conducted using the Pearson correlation test. A *p* value < 0.05 was considered statistical difference.

Because IL-17 is the main cytokine secreted by Th17 cells and ROR-*γ*t is the key transcription factor, while IL-10 is reported to be an important anti-inflammatory cytokine secreted by both Treg and Breg cells and Foxp3 is the main transcription factor expressed in Treg cells, we next measured the mRNA expression levels of these genes to further understand the functional differences in Th17, Treg, and Breg cells between the GD patients and the normal controls. As expected in Figure 2, we observed significantly higher relative IL-17 and ROR-*γ*t mRNA expression levels in the peripheral blood of GD patients than those in healthy controls (mean ± SD: 1.69 ± 0.50 versus 0.75 ± 0.22 (*p* < 0.0001) and 2.04 ± 1.19 versus 0.84 ± 0.52 (*p* = 0.0002), resp.). Circulating relative Foxp3 mRNA expression was significantly lower in the GD group than that in the controls (mean ± SD: 0.61 ± 0.26 versus 1.37 ± 0.43, *p* < 0.0001). However, we did not observe a significant difference in IL-10 mRNA expression between the GD group and the controls (mean ± SD, 0.97 ± 0.64 versus 1.38 ± 1.11, *p* = 0.15). In the correlation analysis, the IL-17 and ROR-*γ*t mRNA levels were positively correlated with circulating Th17 cell proportion, and also the Foxp3 mRNA level was positively correlated with circulating Treg cells, while no correlation was observed between the IL-10 mRNA level and Breg numbers.

### 3.3. The Serum TSAb Activity Was Positively Correlated with the Circulating Th17 Proportion and Negatively Correlated with the CD4^+^Foxp3^+^ Treg and CD19^+^CD1d^hi^CD5^+^ Breg Proportions

**Figure 3:**
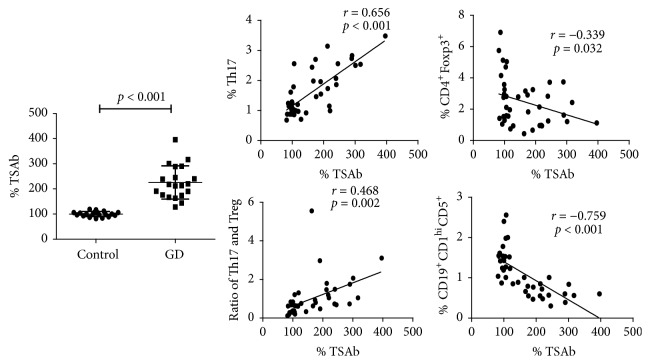
*The levels of serum TSAb activity (%) in healthy controls and patients group and the correlations between serum TSAb activity and circulating Th17, CD4* ^+^ *Foxp3* ^+^ *Treg, and CD19* ^+^ *CD1d* ^*hi*^ *CD5* ^+^ *Breg proportions and the ratio of Th17/Treg*. The levels of serum TSAb activity were shown as mean ± SD. The correlation analysis was conducted using the Pearson correlation test. A *p* value < 0.05 was considered statistical difference.

Due to the different percentages of circulating Th17, Treg, and Breg cells between the GD patients and controls, we next explored the potential correlations of these lymphocyte subsets with TSAb activity. As shown in Figure 3, the serum TSAb activity was positively correlated with the circulating Th17 subset proportion (*r* = 0.656, *p* < 0.001) but negatively correlated with the circulating CD4^+^Foxp3^+^ Treg and CD19^+^CD1d^hi^CD5^+^ Breg subset proportions (*r* = −0.339, *p* = 0.032; *r* = −0.759, *p* < 0.001). Besides, the serum TSAb activity was also significantly positively correlated with the ratio of Th17/Treg (*r* = 0.468, *p* = 0.002).

## 4. Discussion

In this study, we detected a significantly decreased proportion of circulating CD4^+^Foxp3^+^ Treg cells and reduced mRNA expression of the transcription factor Foxp3 in patients with new-onset GD. This result is consistent with our previous study in a murine model [4] and is also supported by many studies, including a recently published report by Li et al. [7, 8, 19]. The decreased circulating Treg cells may be partially explained by the increased apoptosis of Treg cells in GD patients. Nakano et al. detected a greater proportion of apoptotic cells among CD4^+^CD25^+^ Treg cells than among CD4^+^CD25^−^ T cells in the thyroids of patients with autoimmune thyroid diseases, indicating that many Treg cells undergo apoptosis in the thyroids of these patients [20]. Mao et al. demonstrated that the circulating CD4^+^CD25^+^ Treg cells could be induced to undergo apoptosis by the CD11c^−^CD123^hi^ plasmacytoid dendritic cells that predominantly exist in untreated GD patients [19]. Different from our report, Bossowski et al. found a normal proportion of circulating CD4^+^CD25^+^CD127^−^ Treg cells in GD patients [6], possibly because they used a different phenotype to identify Treg cells. In their another study [21], they found that the percentage of CD4^+^Foxp3^+^ Tregs was significantly decreased in untreated GD patients like ours; yet, the proportion of the CD4^+^CD25^+^CD127^−^ T cell subset remained normal. Notably, we also found that the proportion of CD4^+^Foxp3^+^ Treg cells was inversely correlated with TSAb activity, suggesting that the deficient Treg cells in GD patients may lose their capacity to prevent B cells from producing autoantibodies.

Numerous studies have deduced that autoimmune disease development is associated closely with excessive Th17 activity [22]. In support of this likelihood, our study found an increased percentage of Th17 cells and enhanced IL-17 and ROR-*γ*t mRNA expression levels in patients with new-onset GD. In addition, a positive correlation was found between the proportion of circulating Th17 cells and serum TSAb activity, suggesting a proinflammatory role for Th17 cells. These results are also consistent with those of Peng et al. [23]. However, some studies do not support our findings. Bossowski et al. [6] found a reduced percentage of Th17 cells accompanied by a normal percentage of Treg cells in untreated GD patients. Qin et al. [24] found significantly increased circulating IL-17 mRNA expression in patients with Hashimoto's thyroiditis (HT) but not in those with GD. Figueroa-Vega et al. [25] reported increased differentiation of Th17 lymphocytes and enhanced Th17 cytokine expression in the PBMCs of HT patients but not in those of GD patients. Our previous study [4] also found that Tregs and the ratio of Th17/Treg, but not Th17, were modulated in the mouse model of GD. The apparent discrepancies between these studies and our present one may be partially due to the different genetic backgrounds of the subjects involved. Horie et al. [5] once reported that mice with the BALB/c genetic background developed hyperthyroidism regardless of whether the IL-17 gene was knocked out; however, some wild-type but not IL-17 knockout NOD.H-2^h4^ mice experienced hyperthyroidism, indicating that IL-17 functions can depend on the genetic background of the species. Our study, together with other studies conducted in the Chinese population [7, 23], suggests that Chinese patients diagnosed with new-onset GD may have an increased Th17 response in circulation.

In a previous study, we were the first to propose that the imbalance of Th17/Tregs played an important role in GD pathogenesis, and some researchers paid attention. Recently, two studies have reported abnormal ratios of Th17/Tregs in patients with autoimmune thyroid diseases [6, 7]. In our present study, we found that the ratio of circulating Th17/Tregs was significantly higher in GD patients than in normal controls. Actually, apart from the balance of Th1/Th2, the Th17/Treg is becoming a new pair to assess immune disorders. The close relationship between Tregs and Th17 cells has been explored for years. Foxp3, a key Treg transcription factor, could limit the Th17 response by binding to STAT-3, a main factor for Th17 differentiation [26]. Bossowski et al. [6] reported a decreased Th17/Treg ratio in PBMCs from children with GD, while Li et al. [7] found an increased circulating Th17/Treg ratio in GD patients. Here, we suggest that an abnormal Th17/Treg ratio may indicate immune disorder in GD.

Our study also found that the proportion of the CD19^+^CD1d^hi^CD5^+^ Breg subset was significantly decreased in GD patients. Zhang et al. [17] reported that B cells with a phenotype of CD19^+^CD1d^hi^CD5^+^ were increased in the peripheral blood of patients with tuberculosis and had an inhibitory function on Th17 responses. Correale et al. [27] reported that IL-10-producing B cells in helminth-infected multiple sclerosis patients could express high cell surface CD1d levels. Kristensen et al. [9] found that IL-10 could be induced more highly in the CD5^+^ B cell subgroup than in the CD5^−^ subgroup in the peripheral blood of healthy people. Thus, the B cell subset marked by CD1d^hi^ and CD5^+^ should play a regulatory role in humans, and this function may partially depend on IL-10 secretion. Our previous study explored the quantitative changes in the CD19^+^CD1d^hi^CD5^+^ B cell subset in the iodine-induced autoimmune thyroiditis of NOD.H-2^h4^ mice [28] and found a significant decrease in this B cell subset and reduced IL-10 mRNA expression in mouse splenocytes. Here, we found similar results in the blood of GD patients except in terms of IL-10 mRNA expression, which was not significantly decreased, possibly because the real changes of the specific IL-10 produced by CD1d^hi^CD5^+^ B cells are hidden in the changes in the total IL-10 secretion of PBMCs. We also found that the proportion of CD1d^hi^CD5^+^ Breg cells was negatively correlated with serum TSAb activity, suggesting a regulatory role of this phenotype of B cells for TSAb production. To the best of our knowledge, this report is the first concerning CD1d^hi^CD5^+^ Breg cells in GD patients. However, some limitations obviously exist. First, we did not detect the IL-10 production specifically secreted by CD19^+^CD1d^hi^CD5^+^ B cells. Second, it remains unclear how these B cells suppress autoimmunity to demonstrate their regulatory capacity. Unlike in mice with certain gene deficits that have expanded CD19^+^CD1d^hi^CD5^+^ B cell subsets, this B cell subset is extremely rare among the PBMCs of the general population, making it difficult to understand completely [29]. Thus, a specific approach to amplify this B cell subset in human disease is needed.

## 5. Conclusions

This study indicated increased Th17, impaired CD4^+^Foxp3^+^ Treg, elevated Th17/Treg ratios, and a decreased proportion of CD19^+^CD1d^hi^CD5^+^ Breg cells among the PBMCs of Chinese patients with new-onset GD. Studies are needed to further identify the regulatory function of the CD19^+^CD1d^hi^CD5^+^ B cell subset in Graves' disease.

## Figures and Tables

**Table 1:** The clinical characteristics of the participants.

	Healthy control	GD patients
Number	20	20
Age (year)^a^	32.00 ± 6.14	36.65 ± 11.00
Gender (F/M)	13/7	13/7
FT_4_ (pmol/L)^b^	16.0 (11.7–21.9)	63.7 (32.7–77.2)^*∗*^
TSH (mU/L)^b^	1.390 (0.458–3.320)	0.004 (0.004–0.019)^*∗*^
TPOAb (IU/ml)^b^	10.0 (10.0–16.1)	261.0 (10.0–1000.0)^*∗*^
TgAb (IU/ml)^b^	20.0 (20.0–69.6)	20.0 (20.0–793.0)^*∗*^
TSAb (%)^b^	100 (82–119)	216 (128–396)^*∗*^
TPOAb positive (*n*/*n*)	0/20	14/20^*∗*^
TgAb positive (*n*/*n*)	1/20	7/20^*∗*^

^a^Data presented as mean ± SD. ^b^Data presented as median (range). ^*∗*^*p* < 0.05 compared with the healthy control group.
